# Ultrastructural Sperm Flagellum Defects in a Patient With *CCDC39* Compound Heterozygous Mutations and Primary Ciliary Dyskinesia/*Situs Viscerum Inversus*

**DOI:** 10.3389/fgene.2020.00974

**Published:** 2020-08-28

**Authors:** Rossella Cannarella, Eugenia Tiziana Maniscalchi, Rosita Angela Condorelli, Marina Scalia, Giulia Guerri, Sandro La Vignera, Matteo Bertelli, Aldo Eugenio Calogero

**Affiliations:** ^1^Department of Clinical and Experimental Medicine, University of Catania, Catania, Italy; ^2^MAGI EUREGIO, Bolzano, Italy; ^3^Department of Biomedical and Biotechnological Sciences, Section of Biology and Genetics, University of Catania, Catania, Italy

**Keywords:** primary ciliary dyskinesia, Kartagener syndrome, *CCDC39*, *CCDC151*, *situs inversus*, asthenozoospermia

## Abstract

**Introduction:** Primary ciliary dyskinesia (PCD) is a rare autosomal recessive disease characterized by structural or functional motile cilia abnormalities. Up to 40 different genes seem, at the moment, to be involved in the pathogenesis of PCD. A number of ultrastructural defects have also been reported in sperm flagella, but the sperm mitochondrial membrane potential (MMP) has never been described in these cases.

**Aim:** The aim of this study was to report the sperm MMP and ultrastructural abnormalities of the sperm flagella found in a patient with PCD and *situs inversus* (Kartagener syndrome) and its characterization from the genetic point of view.

**Methods:** Transmission electronic microscopy (TEM) analysis was used to evaluate flagella ultrastructure. The genetic testing was performed by next-generation sequencing. Sperm DNA fragmentation and MMP were also evaluated by flow cytometry.

**Results:** We report here the case of an 18-year-old male patient with PCD and *situs inversus* and severe oligo-astheno-teratozoospermia. TEM analysis of his spermatozoa showed an abnormal connecting piece. The mid piece appeared abnormally thickened, with cytoplasmic residue, dysplasia of fibrous sheath, loss of the outer dynein arms (ODAs), truncated inner dynein arms, and supernumerary outer fibers. The percentage of spermatozoa with fragmented DNA was normal, whereas a high percentage of spermatozoa had low MMP, suggesting an altered mitochondrial function. The genetic analysis showed the presence of c.610-2A > G, p.Arg811Cys compound heterozygous mutations in the *CCDC39* gene.

**Conclusion:** The case herein reported suggests that the high percentage of sperm with low MMP may play a role in the pathogenesis of asthenozoospermia in patients with Kartagener syndrome. In addition, we report, for the first time, the missense variant p.Arg811Cys in the *CCDC39* gene in a patient with Kartagener syndrome. Although *in silico* analysis predicts its damaging potential, its clinical meaning remains unclear.

## Introduction

Primary ciliary dyskinesia (PCD) is a heterogeneous and multisystemic disorder characterized by structural and/or functional abnormalities of the motile cilia. It is a rare autosomal recessive disease, whose prevalence varies from 1:10,000 to 1:20,000 people worldwide (Gunes et al., 2018). The clinical features include chronic sinusitis, bronchiectasis, rhinitis, rhinorrhea, and chronic cough, largely due to a defective mucociliar clearance of the respiratory tract (Barbato et al., 2009). About half of PCD patients have *situs inversus*, whose condition is known as Kartagener syndrome (MIM number 244400) (Zariwala et al., 2006).

Infertility is common in male patients with PCD/Kartagener syndrome. It is mainly ascribed to flagella abnormalities, leading to sperm immotility (Gunes et al., 2018). Transmission electron microscopy (TEM) analysis has documented a number of ultrastructural defects at the sperm level, such as dynein arm deficiency (Kordus et al., 2008), abnormal connecting pieces, shortened mid pieces with attenuated mitochondria sheaths, poorly developed annulus, abnormal outer dense fibers, axonema missing the two central microtubules (Yokota et al., 1993; McLachlan et al., 2012), or dysplasia of the fibrous sheath (Moretti et al., 2017).

To date, up to 40 genes have been reported involved in the pathogenesis of PCD/Kartagener syndrome (Kurkowiak et al., 2015; Ji et al., 2017). Most of them encode components of the outer dynein arms (ODAs), radial spokes, inner dynein arms (IDAs), and cytoplasmic pre-assembly factors of axonemal dynein (Ji et al., 2017). In particular, the *CCDC39* gene has been suggested to be involved in the development of PCD with axonemal disorganization and loss of IDA (Merveille et al., 2011; Antony et al., 2013).

To date, few associations have been reported between mutations on the *CCDC39* gene and *situs inversus*. Merveille et al. (2011), described nine patients with Kartagener syndrome with homozygous (consanguineous patients) or compound heterozygous mutations on the *CCDC39* gene. A similar phenotype has been reported in patients with *CCDC39* or *CCDC40* gene mutations (Blanchon et al., 2012).

Sperm mitochondrion is an organelle surrounding the mid piece, wrapped around the axoneme. It represents the energetic source of spermatozoa, producing ATP molecules that supply energy for sperm flagellum beats. The sperm mitochondrial membrane potential (MMP) is a bio-functional parameter that provides information on mitochondrial function. Studies indicate a direct correlation between low MMP and reduced sperm motility (defined asthenozoospermia) (Barbagallo et al., 2020). Asthenozoospermia is a typical trait of Kartagener syndrome. To the best of our knowledge, sperm MMP in patients with Kartagener syndrome has never been reported. The aim of this study was to describe the sperm MMP and the ultrastructural abnormalities of the sperm flagella in a patient with Kartagener syndrome, whose genetic testing showed compound heterozygous mutations of the *CCDC39* gene.

## Case Presentation

LMG is an 18-year-old man who was diagnosed with *situs inversus* when he was 10 and had an episode of abdominal pain. In that occasion, instrumental examination revealed dextrocardia with no congenital heart defects. All the abdominal visceral organs were reversed, in the absence of bowel, spleen, or liver abnormalities. TEM analysis of the respiratory cilia demonstrated PCD with abnormalities in the number and position of peripheral microtubules and of central pair (CP). However, with the exception of a single episode of bronchiolitis at the age of 1 year, the patient’s clinical history did not report respiratory symptoms.

He underwent an andrological counseling at the age of 16 due to a mild pain in the right hemiscrotum. The physical examination showed a normal degree of androgenization, no gynecomastia, and normal penis size and testicular volume. The Valsalva’s maneuver at the scrotal level turned out to be positive on the right. The ultrasound revealed normal testicular volumes (13 ml, bilaterally) and a V degree (according to the Sarteschi’s classification) right varicocele. The sperm analysis documented the presence of azoospermia. Gonadotropins and total testosterone were within the normal range. He then underwent scleroembolization of the right varicocele. At 1-year follow-up, the sperm analysis showed oligo-astheno-teratozoospermia (sperm concentration 5 million/ml; total sperm count 5 million/ejaculate, progressive motility 15%, total motility 46%, normal-shaped spermatozoa 1%).

The patient was the third born of four children. His brother (the firstborn), mother, and father had no morbidity. His sister had Wolf-Parkinson-White syndrome. The patient’s brother was fertile and had fathered a child.

The patient’s spermatozoa were used for scanning electron microscopy (SEM) and TEM analyses. We also evaluated sperm DNA fragmentation and MMP. The genetic testing was carried out in the patient and his family.

## Methods

### Sperm Analysis

Several semen samples were collected by masturbation into a sterile container after 4 days of sexual abstinence and were analyzed immediately after liquefaction. According to the 2010 WHO guidelines, each sample was evaluated for seminal volume, pH, sperm count, progressive motility, morphology, and round cell concentration (World Health Organization [WHO], 2010).

### Assessment of Sperm DNA Fragmentation and Mitochondrial Function

Sperm DNA fragmentation and MMP were evaluated by flow cytometry using an EPICS XL (Becker Coulter, Milan, Italy), as previously reported (La Vignera et al., 2012). DNA fragmentation was determined by terminal deoxynucleotidyl transferase-mediated deoxyuridine triphosphate nick end labeling (TUNEL) staining. The negative control was obtained by not adding terminal deoxynucleotidyl transferase to the reaction mixture, whereas the positive control was obtained by pretreating spermatozoa with 1 g/ml of RNase-free deoxyribonuclease I (Sigma Chemical, St. Louis, MO, United states) at 37°C for 60 min before labeling.

Sperm MMP was evaluated following staining with 5,5′,6,6′-tetrachloro-1,1′,3,3′-tetraethylbenzimidazolylcarbocyanine chloride (JC-1). Briefly, an aliquot containing 10^6^ spermatozoa was incubated with JC-1 (5,5′,6,6′-tetrachloro-1,1′,3,3′-tetraethylbenzimidazolylcarbocyanine iodide) (Space Import Export, Milan, Italy) in the dark for 10 min at a temperature of 37°C. At the end of the incubation period, cells were washed in phosphate buffer saline (PBS) and analyzed.

### Genetic Testing

The DNA of all patients was extracted from peripheral blood using a commercial kit (SAMAG BLOOD DNA Extraction Kit). The proband’s DNA was used for next-generation sequencing (NGS) on a MiSeq Illumina instrument with a custom gene panel designed for CDP. The target regions were enriched by Illumina Nextera Rapid Capture Enrichment kit. The panel comprised the following genes: *DNAI1* (OMIM: 604366), *DNAAF3* (OMIM: 614566), *DNAH5* (OMIM: 603335), *HYDIN* (OMIM: 610812), *NME8* (*TXNDC3*, OMIM: 607421), *DNAH11* (OMIM: 603339), *DNAI2* (OMIM: 605483), *DNAAF2* (KTU, OMIM: 612517), *RSPH4A* (OMIM: 612647), *RSPH9* (OMIM: 612648), *DNAAF1* (*LRRC50*, OMIM: 613190), *CCDC39* (OMIM: 613798), *CCDC40* (OMIM: 613799), *DNAL1* (OMIM: 610062), *CCDC103* (OMIM: 614677), *DNAAF5* (*HEATR2*, OMIM: 614864), *LRRC6* (OMIM: 614930), *CCDC114* (OMIM: 615038), *DRC1* (*CCDC164*, OMIM: 615288), *ZMYND10* (OMIM: 607070), *ARMC4* (OMIM: 615408), *RSPH1* (OMIM: 609314), *DNAAF4* (*DYX1C1*, OMIM: 608706), *C21ORF59* (CFAP298, OMIM: 615494), *CCDC65* (*DRC2*, OMIM: 611088), *SPAG1* (OMIM: 603395), *CCNO* (OMIM: 607752), *CCDC151* (OMIM: 615956), *CENPF* (OMIM: 600236), *RSPH3* (OMIM: 615876), *GAS8* (OMIM: 605178), *DNAJB13* (OMIM: 610263), *TTC25* (OMIM: 617095), *PIH1D3* (OMIM: 300933), *DNAH1* (OMIM: 603332), *DNAH8* (OMIM: 603337), *MCIDAS* (OMIM: 614086), *STK36* (OMIM: 607652). The sequences were mapped on the human reference sequence GRCh38. Pathogenic variations were sought in Human Gene Mutation Database (HGMD professional) and MASTERMIND^1^. Sanger sequencing was used to confirm NGS variants, and it is also used for the study of variant segregation in family members.

### Mutation Analysis

The variants were filtered as follows: (1) variants with minor allele frequency (MAF) of less than 1% in 1000 Genomes^2^, EVS^3^, and GNOMAD^4^ database are considered; (2) Evaluation is focused on coding exons along with flanking ± 15 intronic bases; (3) For synonymous and splicing variants with GMAF/MAX MAF clearly inferior to known frequency of the pathology, the presence on the database was verified, such as The Human Gene Mutation Database (HGMD). The interpretation of variants is produced through the scoring system by the American College of Medical Genetics and Genomics (ACMG) guidelines. All variants related to the phenotype of the patient, except benign or likely benign variants, are reported. The highlighted variants are classified into pathogenetic, probably pathogenic, and of uncertain significance. Bioinformatics tools were used to predict pathogenicity *in silico* (such as SIFT, MutationTaster, PROVEAN, Polyphen2) and to evaluate the evolutionary conservation for missense variants.

### Transmission Electron Microscope Analysis

For SEM, spermatozoa were allowed to settle into a sterile circular polylysine-coated cover glass, fixed with 2.5% glutaraldehyde in 0.12 M PBS overnight at 4°C. After washing with phosphate buffer several times, it was postfixed in 1% buffered osmium tetroxide for 1 h at 4°C, rinsed 2 × 2 min with PBS, dehydrated in a graded ethanol series, dried by critical point technique, and sputtered with 5 nm gold layer using an Emscope SM 300 (Emscope Laboratories, Ashford, United Kingdom). Observations were carried out using a Zeiss field emission scanning electron microscope. For TEM, the sperm sample (2 × 10^6^ million/ejaculate) was fixed with 2.5% glutaraldehyde in 0.12 M PBS for 2 h at 4°C, centrifuged at 1,000 × *g* for 15 min. The pellet was washed three times in phosphate buffer. It was then postfixed in 1% buffered osmium tetroxide for 1 h at 4°C, included in a cloth of fibrin, dehydrated in a graded series of acetone, and embedded in Durcupan ACM (Fluka Chemika-Biochemika, Buchs, Switzerland). Ultrathin sections were cut perpendicularly for the membrane using a Reichert Ultracut E microtome and double stained with uranyl acetate and lead citrate. Observations were carried out using a Hitachi H-7000 transmission electron microscope (Hitachi High-Technologies Europe GmbH, Krefeld, Germany).

### Ethical Approval

This study was conducted at the Division of Andrology and Endocrinology of the teaching hospital “G. Rodolico,” University of Catania (Catania, Italy). The protocol was approved by the internal institutional review board, and informed written consent to participate in the study and to publish was obtained from the participant after full explanation of the purpose and nature of all procedures used. The study has been conducted in accordance with the principles expressed in the Declaration of Helsinki.

## Results

### Transmission Electron Microscope Analysis

**FIGURE 1:**
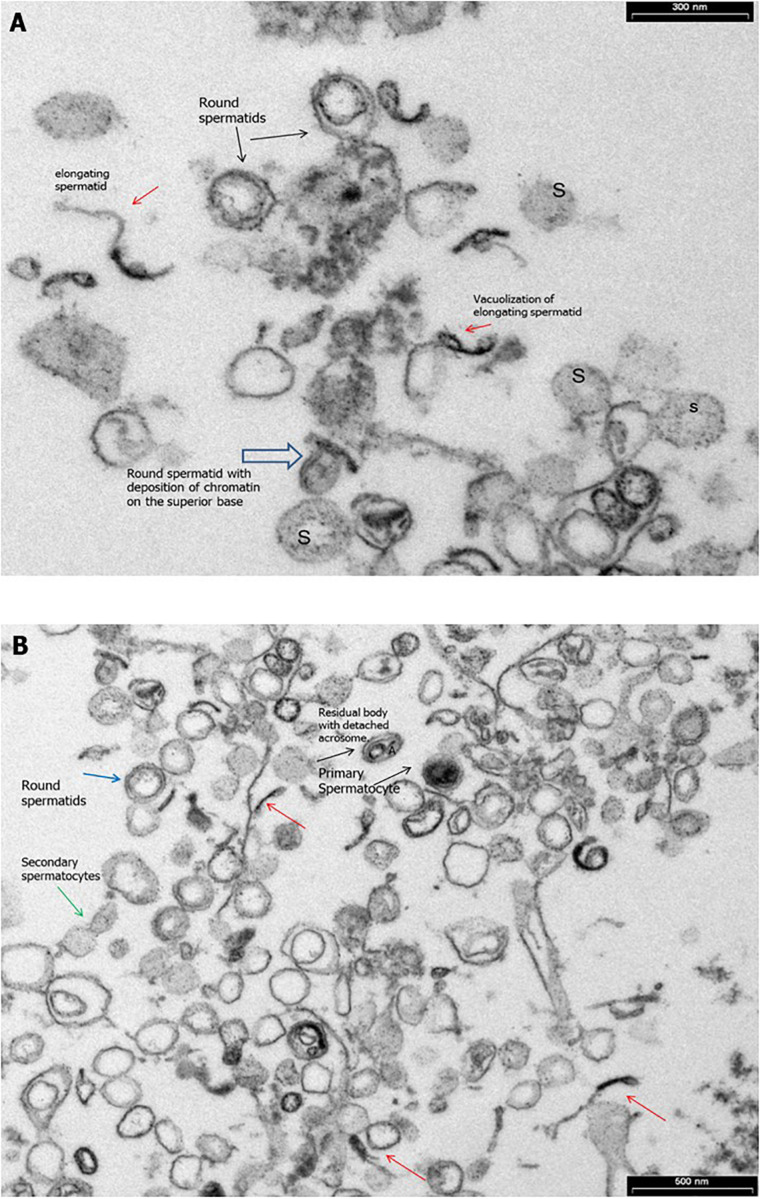
Transmission electron microscopy (TEM) micrographs of ejaculated spermatozoa. **(A)** Numerous elements of the spermatogenic line ranging from elongated spermatids (red arrow) with or without vacuolizations (red arrow) to spermatids round (black arrows) with deposition of chromatin on the superior base (empty arrow) and spermatogonia (S) during the spermiogenesis. Bar = 300 nm. **(B)** TEM micrographs of ejaculated spermatozoa showing numerous elements of the spermatogenic line ranging from elongated spermatids (red arrows) to primary spermatocytes (black arrows) and secondary spermatocytes with cytoplasmic bridge (green arrows). Bar = 600 nm.

**FIGURE 2:**
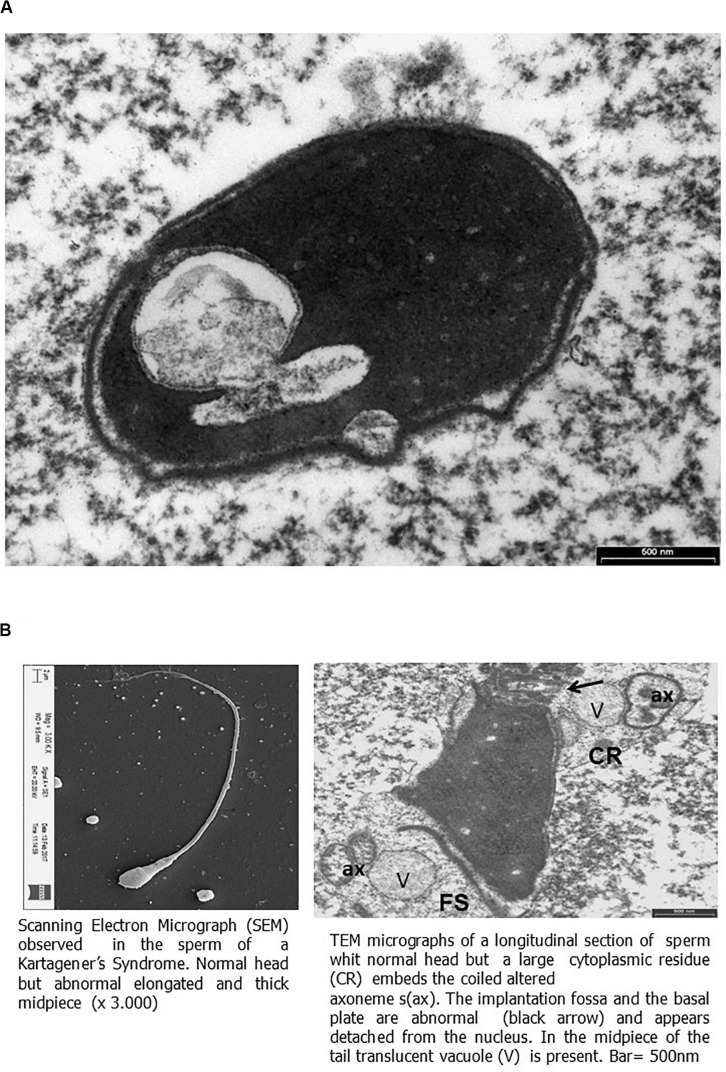
Scanning electron micrograph (SEM) and transmission electron microscopy (TEM) micrographs of ejaculated spermatozoa. **(A)** TEM micrographs of longitudinal section of the sperm head showing a large nuclear vacuole filled with membrane in the nucleus and acrosome with protuberances detached. Bar = 500 nm. **(B)** On the left, SEM micrograph of sperm showing elongated and thick mid piece. On the right, TEM micrographs (as shown by SEM) of a thickened mid piece due to a disorganized fibrous sheath (FS), showing a large cytoplasmic residue (CR) embeds the coiled altered axonemes (ax). The implantation fossa and the basal plate are abnormal (black arrow) and appear detached from the nucleus. In the mid piece of the tail, translucent vacuole (V) is present. Bar = 500 nm.

**FIGURE 3:**
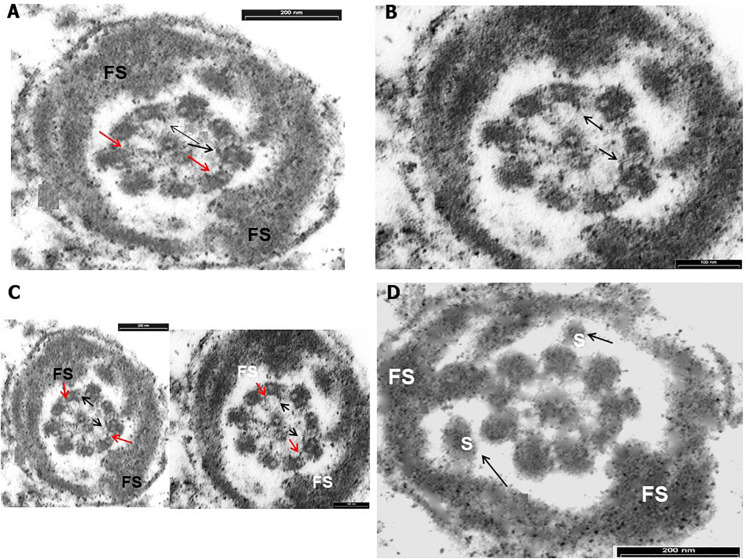
Transverse section through the principal piece of spermatozoa at low magnification. **(A)** Peripheral fibrous sheath (FS) very abundant with diffuse disorganization. Nine peripheral doublet microtubules + a central pair (CP) show the absence of outer dynein arm (ODA) and the presence of inner (red arrows) or truncated inner dynein arm (black arrows). **(B)** Transverse section through the principal piece of spermatozoa at low magnification. Peripheral FS is very abundant with diffuse disorganization. Nine peripheral doublet microtubules + a CP show the absence of ODA and the presence of inner or truncated inner dynein arm (black arrows). **(C)** Transverse section through the principal piece of spermatozoa at low magnification on the left and at higher on the right. Peripheral FS is thickened and very abundant with diffuse disorganization. Nine peripheral doublet microtubules + a CP show the absence of ODA and the presence of inner (red arrows) or truncated inner dynein arm (black arrows). Bar = 200 nm. **(D)** Cross sections of sperm at the principal piece level. The FS appears badly assembled and strongly thickened. Supernumerary outer fibers (S) are present (black arrows). Bar = 200 nm.

Transmission electronic microscopy analysis revealed a wide range of ultrastructural abnormalities in the patient’s spermatozoa, most of which resulting from the widespread immaturity of spermatozoa present in the germ cell population. Figures 1A,B show a number of elements of the spermatogenic line ranging from elongated spermatids (with or without vacuolization) to round spermatids. The few mature spermatozoa present (Figure 2A) showed a nucleus with numerous vacuoles, filled with membrane, within the condensed chromatin and acrosome detached with protuberances. A peculiar sperm tail defect frequently involved the mid pieces of spermatozoa, often showing cytoplasmic residue (CR) and dysplasia of the fibrous sheath (Figure 2A). At the TEM observation, it appeared poorly developed, with few mitochondria and dysplasia of the fibrous sheath, which was badly assembled. It is noteworthy that the misalignment of the connecting piece [spermatozoon lacked the connection between the head and tail with the implantation fossa and basal plate detached from the normal acrosome (A) and nucleus (N)] and large CR embedded the coiled altered axonemes (Figure 2B). Figure 3, at higher magnification, dysplasia of the fibrous sheath and few mitochondria badly assembled were visible. The axonemes of the principal piece showed the presence of a fibrous sheath abnormally abundant with diffuse disorganization. Nine peripheral doublet microtubules were arranged in a circle surrounding a CP of microtubules. The pattern 9 + 2 resulted without ODA and the presence of inner or truncated dynein arm (Figures 3A–C). Other profiles of axonemes always showed the fibrous sheath, being abnormally thick and lacking normal symmetry, and supernumerary outer fibers (Figure 3D).

### Bio-Functional Sperm Parameters

The patient had a high percentage of spermatozoa with low MMP (23.3%; normal values: <11.9%), whereas the percentage of spermatozoa with DNA fragmentation was within the normal range (0.12%, normal values <4.6%).

### Genetic Characterization

The genetic testing was performed by NGS analysis for a custom-made CDP gene panel. We identified two missense mutations in the *CCDC39* gene. The NGS coverage of targeted bases was 161407 with 98.8% covered to at least 10 X. Genetic testing showed that the proband was heterozygous for variants: CCDC39:NM_181426.1:int5:c.610-2A>G(rs756235547), CCDC39:NM_181426.1: ex18: c.2431C>T: NP_852091.1:p.(Arg811Cys) (rs574993914) (Table 1).

**TABLE 1:** Mutations reported in the proband.

Gene	HGVS	Nucleotide change	Amino acid change	Variant identification	Zygosity	Allele origin	gnomAD	*In silico* prediction results	Clinical significance (Varsome)
*CCDC39*	chr3:180659582- 180659582:T/C	NM_181426.1: c.610-2A > G		rs756235547	heterozygous	father	12/143290 (0.00008)	Disease-causing (Mutation Taster)	pathogenic
*CCDC39*	chr3:180616671- 180616671:G/A	NM_181426.1: c.2431C > T	NP_852091.1: p.Arg811Cys	rs574993914	heterozygous	mother	15/214056 (0.00007)	Disease-causing (Mutation Taster) Deleterious (SIFT) Probably damaging (Polyphen)	VUS

**FIGURE 4:**
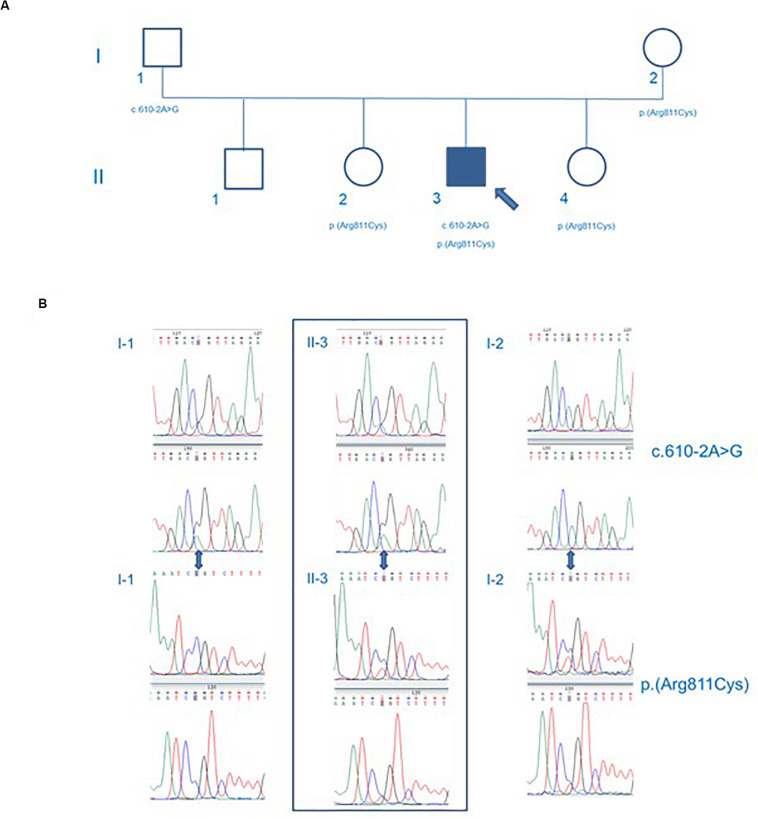
Genealogic tree **(A)** and Sanger sequencing of the CCD39 variants **(B)**. The p.Arg811Cys *CCDC39* gene mutation was found in the mother of the patient, while the c.610-2A > G *CCDC39* gene mutation was found in the father. The patient’s brother inherited no CCDC39 mutations. Both sisters inherited the p.Arg811Cys heterozygote mutation. The patient showed a compound heterozygosity for p.Arg811Cys and c.610-2A > G *CCDC39* gene mutations. Circles represent females. Squares represent males. White forms indicate no phenotypic abnormalities; black ones indicate pathologic phenotypes.

These variants were validated with Sanger sequencing. Moreover, Sanger sequencing of the proband’s healthy parents showed c.610-2A > G missense was inherited from the father (I-1) and p.(Arg811Cys) missense was hereditary from the mother (I-2), thus showing a compound heterozygous state (Figure 4).

**FIGURE 5:**
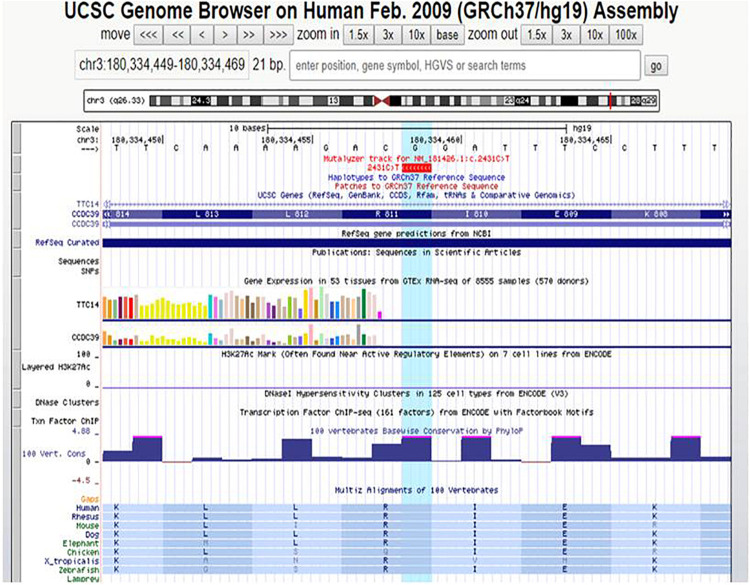
UCSC Genome Browser and amino acid conservation.

**FIGURE 6:**
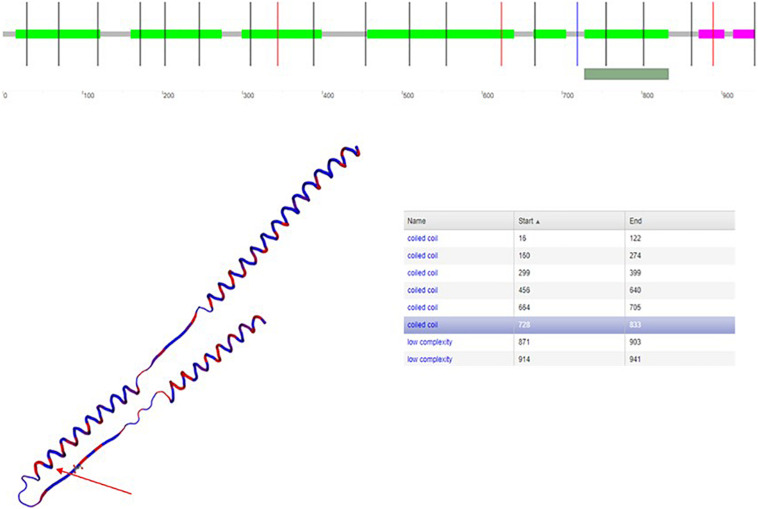
Graphic representation of CCDC39.

The variation c.610-2A > G is a missense of pathogenic significance because it alters an acceptor site splicing, and it has been reported to be associated with PCD (Merveille et al., 2011; Zariwala et al., 2013). The variation p.(Arg811Cys) is a missense that is not listed in ClinVar database; it has never been reported in literature in association with a pathological phenotype. Furthermore, the variant p.(Arg811Cys) is in a conserved amino acid residue during evolution (Figure 5), and it is positioned in a coiled-coil domain in position 811, which causes a change of the amino acid with substitution of hydrophilic (arginine) with a hydrophobic (cysteine) amino acid (Figure 6).

The variant p.(Arg811Cys) is registered on dbSNP database with rs574993914. It shows a very low frequency in scientific reference databases as GnomAD (0.00007), TOPMED (0.00002), and ExAC(0.0001). This variant was predicted to be disease-causing (mutation tasting), deleterious (SIFT), and probably damaging (Polyphen) by *in silico* pathogenicity prediction software.

## Discussion

The human sperm flagellum ultrastructure includes the connecting piece, consisting of distal and proximal centrioles, the mid piece, containing a ring-shaped mitochondrial sheath which surrounds the axoneme, the principal piece, displaying the fibrous sheath around the axoneme, and the end piece, containing only the axoneme (Linck et al., 2016). The latter is set up by microtubules in a typical 9 + 2 pattern with one CP and nine peripheral microtubule doublets. The CP, microtubules, IDAs, nexin, nexin–dynein regulatory complex, ODAs, and radial spokes are essential components of the axoneme (Supplementray Figure S1), all being involved in the regulation and modulation of flagellum movements (Linck et al., 2016). Any alteration in the flagellum ultrastructure may cause severe motility disorders (Sha et al., 2014).

Patients with PCD/Kartagener syndrome have asthenozoospermia, up to total immotility. The sperm number can be normal, despite, as the case herein reported, oligozoospermia (up to azoospermia) has also been reported (Cayan et al., 2001; Westlander et al., 2003; Nunez et al., 2010; Vicdan et al., 2011; McLachlan et al., 2012; Montjean et al., 2015; Mori et al., 2015; TE; Papadimas et al., 1997; Zumbusch et al., 1998; Kay and Irvine, 2000; Peeraer et al., 2004; Kordus et al., 2008; Matsumoto et al., 2010; Hattori et al., 2011; Geber et al., 2012; Kawasaki et al., 2015). Several defects have been described in sperm flagellum ultrastructure. The TEM analysis of the spermatozoa of the patient herein described revealed abnormal CP. The mid piece appeared abnormally thickened, showing CR, dysplasia of fibrous sheath, ODA loss, inner or truncated dynein arm, and supernumerary outer fibers. Similar abnormalities, also including IDA, radial spokes, or CP defects, have been reported in spermatozoa of PCD/Kartagener syndrome patients (Yokota et al., 1993; Kordus et al., 2008; McLachlan et al., 2012). However, in none of these patients data of the genetic testing are available.

Evidence supports the importance of sperm mitochondrial integrity for a normal sperm motility (Barbagallo et al., 2020). Mitochondria is an organelle confined in the mid piece of spermatozoa devoted to oxidative phosphorylation. There is agreement in literature on the role of sperm mitochondria to supply energy for sperm motility (Barbagallo et al., 2020). Sperm MMP is a bio-functional parameter indicating mitochondrial function. Particularly, a low MMP is suggestive of mitochondrial dysfunction, which, in turn, associates with low sperm motility (Paoli et al., 2011; Condorelli et al., 2012). Sperm mitochondrial function has never been evaluated in patients with PCD. We found a high percentage of spermatozoa with low MMP in the patient described in this article. Accordingly, only few mitochondria were found by TEM analysis in our patient, as similarly reported by McLachlan et al. (2012). This suggests that an altered mitochondrial function may contribute to the pathogenesis of asthenozoospermia in PCD patients. Therefore, therapeutic strategies aimed at supporting sperm mitochondrial function may be suggested to possibly improve sperm motility (Mongioi et al., 2016).

Mutations of up to 40 genes encoding for a number of axonemal components (e.g., ODAs, radial spokes, IDAs, or cytoplasmic preassembly factors of axonemal dyneins) have been associated with PCD/Kartagener syndrome (Kurkowiak et al., 2015; Ji et al., 2017; Table 2). This list is rapidly expanding in recent years. The candidate genes have been classified into those that cause ODA defects (e.g., *DNAH5*, *DNAI1*, *DNAI2*, *DNAL1*, *TXNDC3*, and *CCDC114*), considered to be involved in most cases, and those encoding for radial spoke head causing CP abnormalities (*RSPH4A* and *RSPH9*) and genes involved in the assembly and transport of dynein arms which can cause both IDA and ODA defects (e.g., *DNAAF1/LRRC50*, DNAAF2/KTU, *DNAAF3/PF22*, *CCDC103*, *HEATR2*, and *LRRC6*) (Antony et al., 2013). Mutations of *CCDC39* and *CCDC151* genes have been reported in PCD/Kartagener syndrome patients only recently.

**TABLE 2:** Genes involved in primary ciliary dyskinesia.

OMIM phenotype	Gene (MIM number)	Chromosomal locus	Inheritance	Gene name	Biallelic pathogenetic variants in unrelated affected patients*
PCD, 1	*DNAI1* (604366)	9p13.3	AR	Dynein intermediate chain 1, axonemal	2–10%
PCD, 2	*DNAAF3* (614566)	19q13.42	AR	Dynein assembly factor 3, axonemal	<1%
PCD, 3	*DNAH5* (603335)	5p15.2		Dynein heavy chain 5, axonemal	15–29%
PCD, 5	*HYDIN* (610812)	16q22.2	AR	Hydrocephalus-inducing protein homolog	<1%
PCD, 6	*NME8* (*TXNDC3*) (607421)	7p14.1	AR	Thioredoxin domain-containing protein 3	<1%
PCD, 7	*DNAH11* (603339)	7p15.3	AR	Dynein heavy chain 11, axonemal	6–9%
PCD, 9	*DNAI2* (605483)	17q25.1		Dynein intermediate chain 2, axonemal	2%
PCD, 10	*DNAAF2* (*KTU*) (612517)	19q21.3		Protein kintoun	<1–2%
PCD, 11	*RSPH4A* (612647)	6q22.1		Radial spoke head-like protein 3	1–2%
PCD, 12	*RSPH9* (612648)	6p21.1		UF0685 protein C6orf206	<1%
PCD, 13	*DNAAF1* (*LRRC50*) (613190)	16p23.3-q24.1	AR	Dynein assembly factor 1, axonemal	1–2%
PCD, 14	*CCDC39* (613798)	3q26.33	AR	Coiled-coil domain-containing protein 39	<4%
PCD, 15	*CCDC40* (613799)	17q25.3		Coiled-coil domain-containing protein 40	3–4%
PCD, 16	*DNAL1* (610062)	17q24.3	AR	Dynein light chain 1, axonemal	<1%
PCD, 17	*CCDC103* (614677)	17q21.31	AR	Coiled-coil domain-containing protein 103	<4%
PCD, 18	*DNAAF5* (*HEATR2*) (614864)	Unknown	AR	HEAT-repeat containing protein 2	<1%
PCD, 19	*LRRC6* (614930)	8q24.22	AR	Protein TILB homolog	1%
PCD, 20	*CCDC114* (615038)	19q13.33	AR	Coiled-coil domain-containing protein 114	<2%
PCD, 21	*DRC1* (*CCDC164*) (615288)	2p23.3	AR	Dynein regulatory complex protein 1	<1%
PCD, 22	*ZMYND10* (607070)	3p21.31	AR	Zinc finger MYND domain containing protein 2	<2–4%
PCD, 23	*ARMC4* (615408)	10p12.1	AR	Protein TILB homolog	1%
PCD, 24	*RSPH1* (609314)	21q22.3	AR	Radial spoke head 1 homolog	2%
PCD, 25	*DNAAFS* (*DYX1C1*) (608706)	15q21.3	AR	Dyslexia susceptibility 1 candidate gene 1 protein	<1%
PCD, 26	*C21ORF59* (615494)	21q22.11	AR	UPF0769 protein C21orf59	
PCD, 27	*CCDC65* (*DRC2*) (611088)	12q13.12	AR	Coiled-coil domain-containing protein 65	<1%
PCD, 28	*SPAG1* (603395)	8q22.2	AR	Sperm-associated antigen 1	<4%
PCD, 29	*CCNO* (607752)	5q11.2	AR	Cyclin-O	<1%
PCD, 30	*CCDC151* (615956)	19q13.2	AR	Coiled-coil domain-containing protein 151	<3%
PCD, 31	*CENPF* (600236)	1q41	AR	Centromere protein F	<1%
PCD, 32	*RSPH3* (615876)	6q25.3	AR	Radial spoke head protein 3 homolog	<1%
PCD, 33	*GAS8* (605178)	16q24.3	AR	Growth arrest specific 8	
PCD, 34	*DNAJB13* (610263)	11q13.4		DnaJ heat shock protein family (Hsp40) member B13	
PCD, 35	*TTC25* (617095)	17q21.2	AR	Tetratricopeptide repeat domain 25	
PCD, 36, X-linked	*PIH1D3* (300933)	Xq22.3	XLR	PIH1 domain containing 3	9.5% (3/32)
PCD, 37	*DNAH1* (603332)	3p21.1	AR	Dynein heavy chain 1, axonemal	
PCD**	*DNAH8* (603337)	6p21.2		Dynein heavy chain 8, axonemal	<1%
Reduced generation of multiple motile cilia	*MCIDAS* (614086)	5q11.2		Multicilin	<1%
PCD**	*STK36* (607652)	2q35		Serine/threonine kinase 36	

PCD: Ciliary dyskinesia, primary; AR, autosomal recessive; AD, autosomal dominant; XLR, X-linked. *Estimate of all PCD, GeneReview, http://www.ncbi.nlm.nih.gov/books/NBK1122/, Rev. September, 2015. **HGMD (The Human Gene Mutation Database) Reported phenotype.

The *CCDC39* gene, mapping in the 3q26.33 chromosome and consisting of 20 exons, encodes for the coiled-coil domain-containing protein 39 that is expressed in human nasal brushing, lung, and testis (Merveille et al., 2011). It is required for the correct assembly of the DNALI1-containing IDA complexes, the DRC, and radial spokes in human and dogs. Accordingly, TEM analysis performed on respiratory cells from Old English Sheepdogs with *situs inversus* and respiratory symptoms revealed defective IDAs, nexin links, and radial spokes in CCDC39-deficient cilia. No apparent deleterious effect on ODAs has been observed, thus suggesting that *CCDC39* mutations alter only the internal part of the axoneme (Merveille et al., 2011). Mutations of *CCDC39* gene have already been reported in PCD patients (Table 3). Blanchon et al. (2012) reported sperm flagellar ultrastructure abnormalities similar to those found in the ciliary cells, mainly consisting of IDA defects and axonemal disorganization. Antony et al. (2013) reported similar ultrastructural abnormalities. Clinically, these patients were positive for respiratory symptoms, such as neonatal respiratory distress, chronic cough, bronchitis, sinusitis, rhinorrhea, and recurrent respiratory tract infections (Antony et al., 2013). More recently, a retrospective study on 81 Chinese patients with PCD reported mutations of the *CCDC39* gene in 7.8% of cases (5/64). The remaining patients were diagnosed with mutations of other genes, such as *DNAH11*, *HYDIN*, *DNAH5*, *DNAH1*, *CCNO*, and others (Guan et al., 2020). The *CCDC39* gene was one among those with the highest frequency of mutations reported in this study. A high prevalence of chronic wet cough, recurrent sinusitis, bronchiectasis (that also the patient reported in the present article had), and neonatal distress was reported in this cohort (Guan et al., 2020). In contrast, *CCDC39* mutations were found only in one case in 46 Turkish patients with genetically diagnosed PCD (2.2%) (Emiralioğlu et al., 2020).

**TABLE 3:** *CCDC39* and CCDC151 gene mutations reported in patients with primary ciliary dyskinesia.

Gene	Allele 1	Location	Effect	Allele 2	Location	Effect	References
CCDC39	c.2190delA p.Glu731AsnfsX31			c.2190delA p.Glu731AsnfsX31			Blanchon et al., 2012
	c.1072delA p.Thr358GlnfsX3			c.1072delA p.Thr358GlnfsX3			
	c.357 + 1G > C essential splice site	Intron 3	Splice	c.357 + 1G > C essential splice site	Intron 3	Splice	
	c.357 + 1G > C essential splice site	Intron 3	Splice	c.2357_2359delinsT p.Ser786IlefsX33			
	c.357 + 1G > C essential splice site	Intron 3	Splice	C.1848T > G p.Tyr616X			
	c.610-2A > G c.2431C > T			c.2483_2484delTT p.Leu828ProfsX2			
	c.2347_2351delTTTCA p.Phe783ThrfsX3			c.2347_2351delTTTCA p.Phe783ThrfsX3			
	c.802_808dupTTTTTCG p.Glu270ValfsX5			c.2040_2043delTTTG c.Cys680TrpfsX15			
	c.2357_2359delonsT p.Ser786IlefsX33			c.1167 + 1261A > G p.Glu390SerfsX6			
	c.2577C > A p.Tyr859X			c.2577C > A p.Tyr859X			
	c.216_217delTT p.Cys73GlnfsX6			c.216_217delTT p.Cys73GlnfsX6			
	c.1035-3C > G			c.1035-3C > G			
	c.1714C > T p.Arg562X			c.1714C > T p.Arg562X			
	c.1363-3delC			c.1781C > T p.Thr594Ile			
	c.1486_1487insA p.Ser496Tyrfs15	Exon 11	fs	c.2159-2A > G essential splice site	Intron 15	Splice	Antony et al., 2013
	c.2596G > T p.Glu866	Exon 19	Non-sense	c.2596G > T p.Glu866	Exon 19	Non-sense	
	c.357 + 1G > C essential splice site	Intron 3	Splice	c.151C > T p.Arg51	Exon 2	Non-sense	
	c.1795C > T p.Arg599	Exon 13	Non-sense	c.1795C > T p.Arg599	Exon 13	Non-sense	
	c.2039_2040delGT p.Cys680Phefs9	Exon 15	fs	c.526_527delCT p.Leu176Alafs10	Exon 5	fs	
	c.664G > T p.Glu222	Exon 6	Non-sense	c.526_527delCT p.Leu176Alafs10	Exon 5	fs	
	c.2245G > T p.Glu749	Exon 16	Non-sense	c.2245G > T p.Glu749	Exon 16	Non-sense	
	c.1450delA p.Ile484Leufs47	Exon 11	fs	c.357 + 1G > C essential splice site	Intron 3	Splice	
	c.830_831delCA p.Thr277Argfs3	Exon 7	fs	c.830_831delCA p.Thr277Argfs3	Exon 7	fs	
	c.610-2A > G c.2431C > T			c.610-2A > G c.2431C > T			Merveille et al., 2011

*Fs, frameshift.*

The patient described in the present study showed compound heterozygous mutations of the *CCDC39* gene (c.610-2A > G and p.Arg811Cy). To the best of our knowledge, the p.Arg811Cy *CCDC39* mutation has not been reported so far. As already discussed, the patient sperm flagella showed IDA defects that may be ascribed to *CCDC39* mutations.

We report here a missense *CCDC39* gene mutation (p.Arg811Cys) in compound heterozygous with a pathogenetic mutation (c.610-2A) that has already been reported in association with PCD. The p.Arg811Cys mutation has very low allele frequency, in particular 15/210928 in GnomAD, 2/125568 in TOPMED, 4/63054 in ExAC, and 1/5008 in 1000G. This mutation was predicted to be “probably_damaging,” “deleterious,” and “disease-causing” by the bioinformatics software Polyphen, SIFT, and MutationTaster, respectively. Although no animal study has been performed to definitively confirm its pathogenic role (in association with the c.610-2A mutation), the importance of this novel mutation in determining the phenotype cannot be excluded.

Sperm DNA fragmentation rate has been reported only once in patients with PCD. DNA damage was found in a patient with Kartagener syndrome and recurrent fertilization failure (Nunez et al., 2010). The authors concluded that, in addition to the abnormal sperm motility, the patient infertility was likely due to a high level of non-reparable sperm DNA damage. In contrast, we found a normal sperm DNA fragmentation rate. This suggests a lack of ultrastructural flagellum defects *per se* on DNA integrity. As such, the main pathogenic mechanism of male infertility in PCD/Kartagener syndrome is asthenozoospermia, as confirmed by assisted reproductive technique positive outcome (McLachlan et al., 2012).

In conclusion, the case herein reported supports the role of the high percentage of spermatozoa with low MMP in the pathogenesis of asthenozoospermia in this patient with Kartagener syndrome. In addition, we report, for the first time, the missense variant p.Arg811Cys in the *CCDC39* gene in a patient with Kartagener syndrome. Although it is predicted by *in silico* analysis capable of changing a conserved amino acid residue during evolution, with substitution of hydrophilic (arginine) with a hydrophobic (cysteine) amino acid, its clinical relevance remains definitively unclear and needs more studies.

## Data Availability Statement

The datasets generated for this study are available on request to the corresponding author.

## Ethics Statement

Ethical review and approval was not required for the study on human participants in accordance with the local legislation and institutional requirements. Written informed consent to participate in this study was provided by the participants’ legal guardian/next of kin.

## Author Contributions

RC and AC: project managers and writing of the manuscript. RAC and SL: supervision. EM, GG, and MB: genetic assays. MS: sperm assay. All authors contributed to the article and approved the submitted version.

## Conflict of Interest

The authors declare that the research was conducted in the absence of any commercial or financial relationships that could be construed as a potential conflict of interest.

## Footnotes

1 www.mastermind.genomenon

2 http://www.1000genomes.org/home

3 http://evs.gs.washington.edu/EVS/

4 https://gnomad.broadinstitute.org/

## Supplementary Material

FIGURE S1 Schematic representation of the flagellum structure. The human sperm flagellum ultrastructure includes the connecting piece, which consists of distal and proximal centrioles, the mid piece, containing a ring-shaped mitochondrial sheath which surrounds the axoneme, the principal piece, displaying the fibrous sheath around the axoneme, and the end piece, containing only the axoneme. The latter is set up by microtubules in a typical 9 + 2 pattern with one central pair and nine peripheral microtubule doublets. The central pair, microtubules, inner dynein arms, nexin, nexin–dynein regulatory complex, outer dynein arms, and radial spokes are essential components of the axoneme.

## Abbreviations

CP: central pair
CR: cytoplasmic residue
IDA: inner dynein arms
ODA: outer dynein arms
PCD: primary ciliary dyskinesia
SEM: scanning electron microscopy
TEM: transmission electron microscopy.
