# Supplementary figures and images for: Ultrastructural Sperm Flagellum Defects in a Patient With CCDC39 Compound Heterozygous Mutations and Primary Ciliary Dyskinesia/Situs Viscerum Inversus
Source: Front Genet. 2020 Aug 28;11:974. doi: 10.3389/fgene.2020.00974 (PMC7483550; doi:10.3389/fgene.2020.00974)

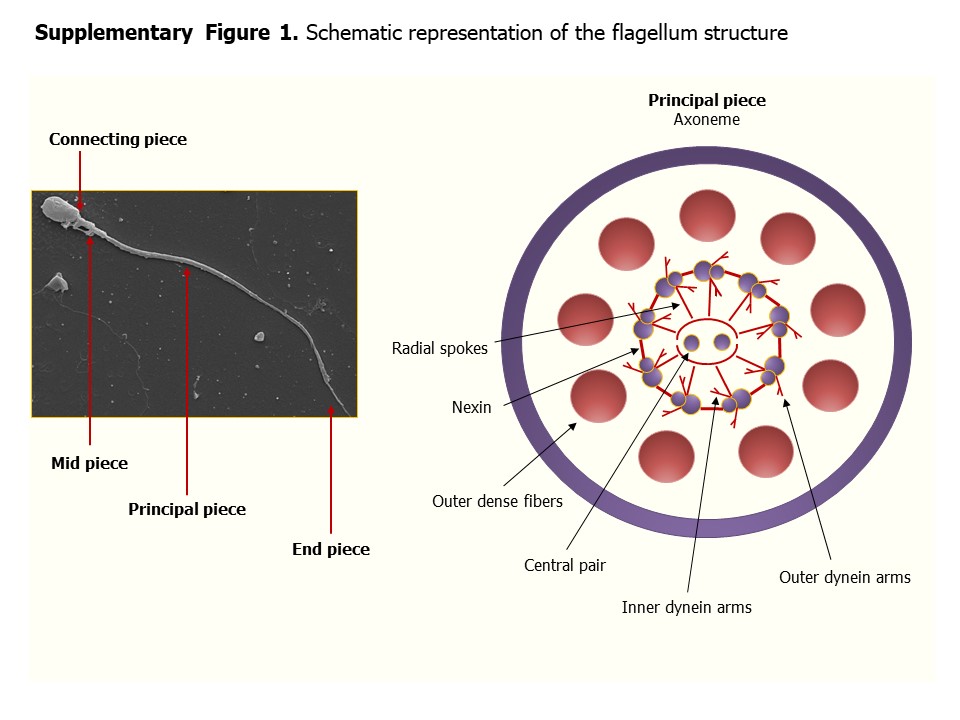

Supplement: FIGURE S1 — Schematic representation of the flagellum structure. The human sperm flagellum ultrastructure includes the connecting piece, which consists of distal and proximal centrioles, the mid piece, containing a ring-shaped mitochondrial sheath which surrounds the axoneme, the principal piece, displaying the fibrous sheath around the axoneme, and the end piece, containing only the axoneme. The latter is set up by microtubules in a typical 9 + 2 pattern with one central pair and nine peripheral microtubule doublets. The central pair, microtubules, inner dynein arms, nexin, nexin–dynein regulatory complex, outer dynein arms, and radial spokes are essential components of the axoneme. [file Image_1.jpeg]
